# Supplementary material for: The α-dystroglycan N-terminus is a broad-spectrum antiviral agent against SARS-CoV-2 and enveloped viruses
Source: Antiviral Res. Author manuscript; Available in PMC 2024 Nov 13. (PMC7616797; doi:10.1016/j.antiviral.2024.105837)
Supplement: Multimedia component 1 [file EMS199604-supplement-Multimedia_component_1.docx]

**Supplementary data**

**Figure S1**

**
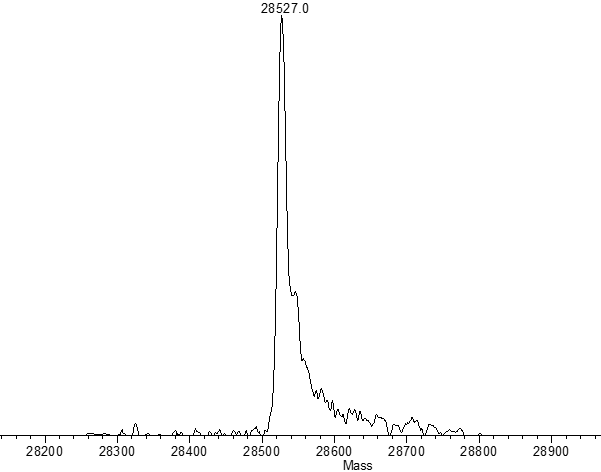

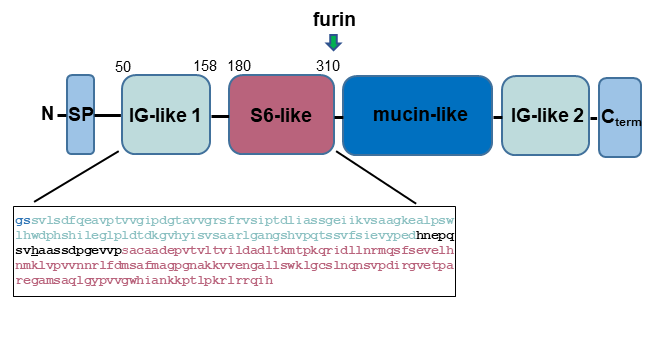
**

**B**

**A**

**Fig. S1. Recombinant α-DGN properties.** (**A**) Domain structure of mouse α-DG (*m*α-DG). SP: signal peptide, Ig-like: immunoglobulin-like domain, S6: ribosomal protein S6-like domain, arrow: furin cleavage site, mucin-like: highly glycosylated central domain of α-DG, C-term: C-terminal portion. The amino acid sequence of the N-terminal domain (α-DGN) as cloned and expressed in this study, is indicated below the cartoon. The sequence of the Ig-like domain is indicated in cyan, the sequence of the S6-like domain is indicated in magenta. The His residue that replaced the original Arg166 in order to stabilize the recombinant product is in black underlined. (**B**) Electrospray Ionization Liquid Chromatography Mass Spectrometry profile of *m*α-DG. The recombinant product runs as a single, sharp peak with a MW of 28527Da, matching the MW calculated based on the amino acid sequence (28528Da).

**
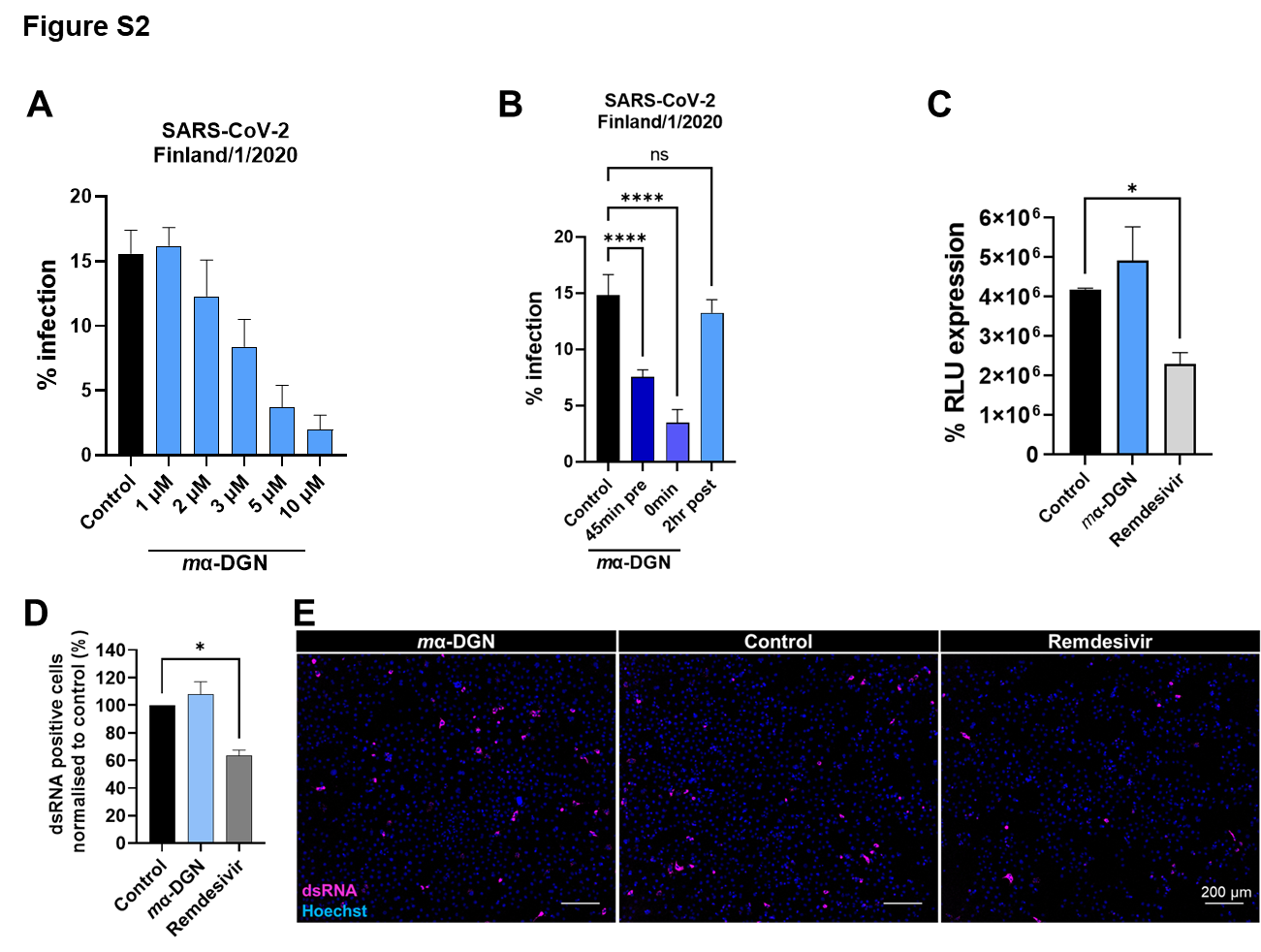
**

**Fig. S2. *m*α-DGN inhibitory activity against SARS-CoV2.** (**A**) Dose dependent inhibitory activity of *m*α-DGN (1 µM-10 µM) against SARS-CoV-2 infection in HEK293T cells expressing human ACE2 and TMPRSS2. The graph shows the % of infection by SARS-CoV-2 (means + SD, n=3). (**B**) Inhibitory activity of *m*α-DGN when pre-incubated with SARS-CoV-2 for 45 min, added at the same time as SARS-CoV-2 or added 2 h post infection with SARS-CoV-2. After a 16 h incubation infection was determined by viral N protein staining. Results are represented as % infection (means + SD). (**C**) Expression levels of Relative luminescence units (RLU) in SARS-CoV-2 replicon RNA transfected VTN cells treated for 18 h with 10 μM *m*α-DGN, vehicle control or 1 μM remdesivir. (**D**) VTN cells transfected with SARS-CoV-2 replicon RNA and treated with 10 μM mα-DGN, vehicle control or 1μM Remdesivir were fixed after 18 h incubation and stained for dsRNA. The graph shows the percentage of dsRNA positive cells after *m*α-DGN, or Remdesivir treatment normalized to the vehicle control treated cells. (**E**) Representative immunofluorescence images of dsRNA (magenta) positive cells after treatment with *m*α-DGN, vehicle control or Remdesivir. Scale bar: 200 µm.

**Figure S3**

**
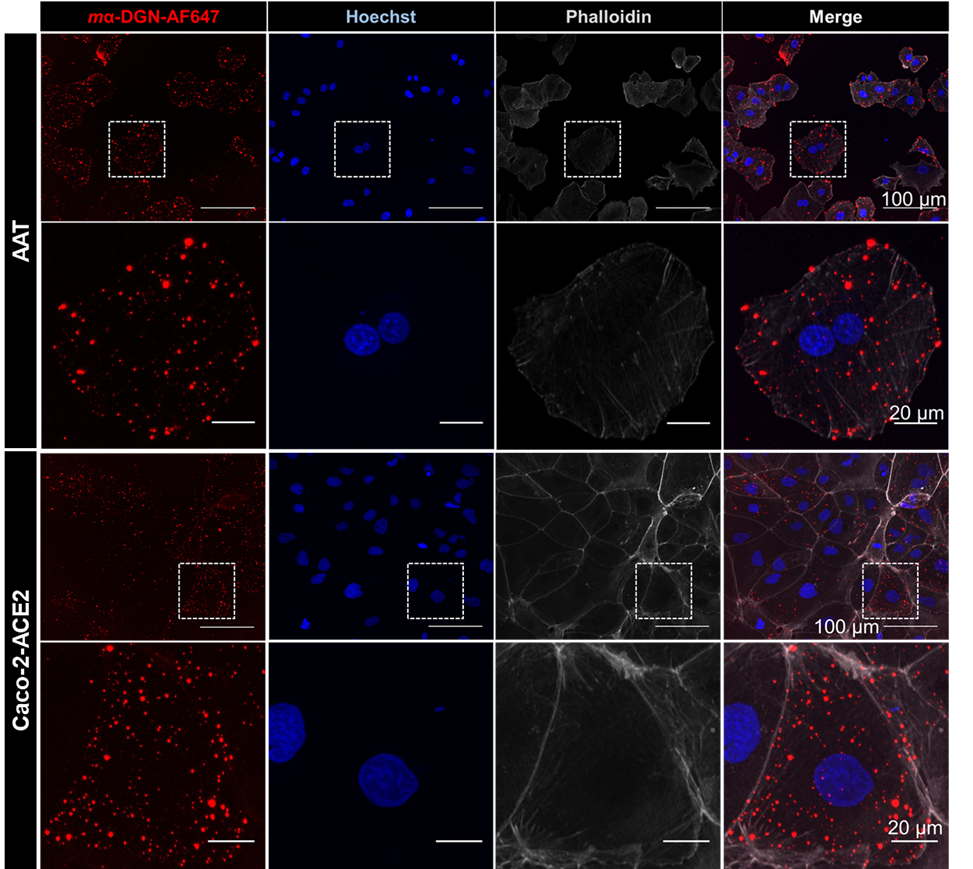
**

**A**

**B**

**Fig. S3. α-DGN binds to different cell lines.** (**A**) A549-ACE2-TMPRSS2 (AAT) or (**B**) Caco-2-ACE2 cells were incubated with 4 µM Alexa Fluor-647 labelled *m*α-DGN for 45 min in the cold and fixed. Nuclei was stained with Hoechst (blue), actin with phalloidin-AF596 (grey) and *m*α-DGN Alexa Fluor-648 is shown in red. Scale bars, 100 µm and 20 µm in the magnified panels. In both panels, the highlighted square regions in the upper image rows were enlarged in the lower image rows.

**Figure S4**

**
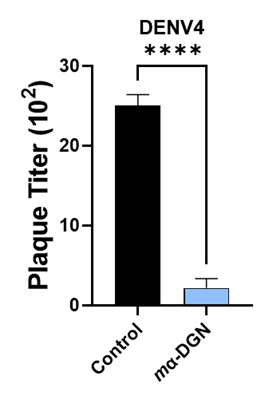
**

**Fig. S4. α-DGN inhibits DENV4 infection of human primary monocytes.**  Human CD14+ monocytes were pre-treated with *m*α-DGN for 2 h prior to infection with DENV4 at an MOI of 10. Inoculum was washed off after 2 h and fresh *m*α-DGN added. After 48 h supernatant was harvested and the viral titre determined by plaque assay. Results are represented as plaque titres (10^2^) of n=6 replicates (means + SD).
